# Co-Infection with *Plasmodium vivax* and COVID-19 in Thailand

**DOI:** 10.3390/tropicalmed7080145

**Published:** 2022-07-22

**Authors:** Parat Boonyarangka, Kittijarankon Phontham, Sabaithip Sriwichai, Kamonporn Poramathikul, Krit Harncharoenkul, Worachet Kuntawunginn, Napat Maneesrikhum, Sarayouth Srisawath, Chanida Seenuan, Chattakorn Thanyakait, Kanjana Inkabajan, Suda Pludpiem, Kingkan Pidtana, Samandra Demons, Brian Vesely, Mariusz Wojnarski, John S. Griesenbeck, Michele Spring

**Affiliations:** 1 Department of Bacterial and Parasitic Diseases, US Army Armed Forces Research Institute of Medical Sciences, Bangkok 10400, Thailand; paratb.ca@afrims.org (P.B.); kittijarankonp.ca@afrims.org (K.P.); sabaithips.fsn@afrims.org (S.S.); kamonpornp.fsn@afrims.org (K.P.); krith.fsn@afrims.org (K.H.); worachetk.fsn@afrims.org (W.K.); kingkanp.ca@afrims.org (K.P.); samandra.demons.mil@afrims.org (S.D.); brian.vesely.mil@afrims.org (B.V.); mariusz.wojnarski.mil@afrims.org (M.W.); john.griesenbeck.mil@afrims.org (J.S.G.); 2 Suan Phueng Hospital, Ratchaburi 70180, Thailand; hut_alone@hotmail.com (N.M.); sarayouth.gun@gmail.com (S.S.); chanida.see19@gmail.com (C.S.); sphos_boss01@hotmail.com (C.T.); pukink@gmail.com (K.I.); 3 Division of Health Promotion and Prevention, Suan Phueng District Health Office, Ratchaburi 70180, Thailand; sudapladpeiym@gmail.com; 4 The Henry M. Jackson Foundation for the Advancement of Military Medicine, Inc., Bethesda, MD 20817, USA

**Keywords:** co-infection, malaria, vivax, COVID-19, Thailand

## Abstract

With the emergence of SARS-CoV-2, healthcare systems not only had to address the pressing clinical needs of the COVID-19 pandemic but anticipate the effect on and of other conditions and diseases. This was of particular concern in areas of the world endemic with malaria, a disease which takes hundreds of thousands of lives each year. This case report from Thailand describes a 25-year-old man diagnosed with *Plasmodium vivax*, who was then found to be co-infected with COVID-19. Both conditions can have overlapping acute febrile illness symptoms which may delay or complicate diagnoses. He had no prior history of malaria and had received two vaccinations against COVID-19. His clinical course was mild with no pulmonary complications or oxygen requirement, and he responded well to treatments for both conditions. Three months after cure, he again contracted COVID-19 but did not experience any *P. vivax* relapse. Review of the available literature produced less than 10 publications describing co-infections with *P. vivax* and COVID-19; nonetheless, in endemic areas, vigilance for both diseases should continue, as co-infections could significantly alter the course of clinical management and prognosis as well as affect the healthcare staff caring for these patients.

## 1. Introduction

In December 2019, Coronavirus Disease 2019 (COVID-19) emerged in Wuhan, China and has since reached pandemic proportions [1]. As of 4 March 2022, there have been 440,807,756 confirmed cases of COVID-19, including 5,978,096 deaths [2]. At the start of the pandemic, officials at the World Health Organization (WHO) expressed concerns that COVID-19, in addition to the significant morbidity on its own, could have dramatic detrimental impacts if co-infections with malaria were to occur, as well as overload health systems and interfere with malaria control efforts [3]. In the first year of the pandemic (2020), malaria cases worldwide increased to 241 million from 227 million in 2019 [4], associated mainly with the disruption to services and the suspension of vector control activities. Combining data from the WHO South-East Asia and Western Pacific Regions from 2020, there were an estimated 400,000 additional cases reported compared to 2019 [4]. 

Even with widely different modes of transmission, malaria is transmitted by Anopheles spp. mosquitoes while COVID-19 is caused by a respiratory viral pathogen, there is an overlap in clinical presentations with acute febrile illness symptoms such as fever, headache, fatigue, and myalgia [5]. As co-infection with malaria and COVID-19 is rarely reported, it is challenging to the differential diagnosis of malaria infection and COVID-19 in malaria-endemic areas. Physicians need to be aware of the co-infection which can occur. This could raise the risk of misdiagnosis, which can decrease the risk of morbidity and mortality due to delayed treatment, as well as have potential impacts on healthcare workers who may be unwittingly exposed to COVID-19 if malaria is initially suspected. Intensive upswings in the use of personal protective equipment (PPE), such as masks, face shields, and gowns, for any encounter with a febrile patient during the pandemic has been one method by which health visits were more safely conducted, and in Thailand, strict policies were implemented on travel and social distancing. These measures appear to be largely successful. To the best of our knowledge, this is the first case report of malaria and COVID-19 co-infection in Thailand.

## 2. Case Report

**Figure 1:**
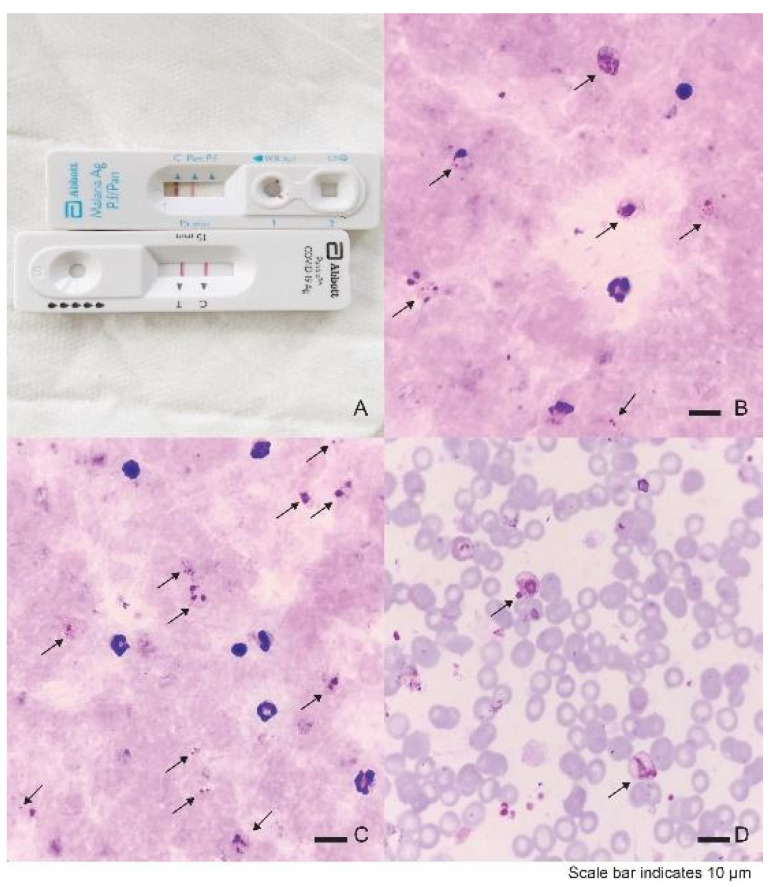
Malaria and COVID-19 diagnostic test results. (**A**) A malaria rapid diagnostic test (upper) was Pan-*Plasmodium*-positive and a SARS-CoV-2 antigen test (lower) was positive. (**B**,**C**) Thick blood film showing several stages of *P. vivax* (arrows), magnification ×1000, Giemsa’s stain. (**D**) Thin blood film showing infected red blood cell with *P. vivax* (arrows), magnification ×1000, Giemsa’s stain.

**Figure 2:**
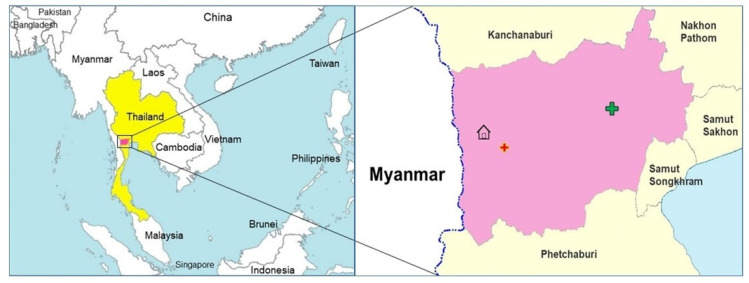
The map display of the patient’s house located in Suan Phueng District, Ratchaburi, Thailand, where the red and green crosses indicate the malaria post and district hospital, respectively.

In November 2021, a 25-year-old Thai man presented to a community malaria post complaining of fever, chills, generalized arthralgia, and headache for five days. A malaria rapid diagnostic test (SD BIOLINE Malaria Ag P.f/P.v, Abbott Diagnostics, Korea) was Pan-*Plasmodium*-positive only (Figure 1), i.e., positive for the genus Plasmodium but a non-falciparum species; thus, the infection was presumed to be *Plasmodium vivax* (*P. vivax*), a relapsing malaria with a 94% prevalence in Thailand [6]. Given this diagnosis, he met the initial eligibility criteria to enroll in a *P. vivax* study being conducted by the Armed Forces Research Institute of Medical Sciences (AFRIMS) in collaboration with the district government hospital and district public health teams in Suan Phueng District, Ratchaburi Province, Thailand (Figure 2).

Prior to enrollment and study participation, the patient was interviewed regarding COVID-19 symptoms and risk factors using the questionnaire from Suan Phueng Hospital, the Ministry of Public Health (MoPH) district-level hospital. He denied having cough, fatigue, and difficulty breathing, as well as any contact with confirmed COVID-19-positive cases or travel/visits to crowded public areas (malls, public transport, etc.) over the past two weeks. He worked at a small, rural resort hotel outside the village, which had temporarily closed due to some employees testing positive for SARS-CoV-2. The subject himself had been tested by a rapid SARS-CoV-2 antigen test two weeks prior and was negative. He then began to travel to the forest to collect mushrooms that would be sold locally. He had received two SARS-CoV-2 vaccinations, completing the course six weeks prior: the Sinovac-Coronavac COVID-19 vaccine for the first dose, followed by the Oxford-AstraZeneca COVID-19 vaccine (Covishield) as the second dose. He appeared in good health, had no overt signs or symptoms, and was able to comfortably converse with the team and answer all questions. A SARS-CoV-2 antigen test (Panbio COVID-19 Ag Rapid Test Device, Abbott, Germany) was required prior to study participation; therefore, a nasopharyngeal swab was collected and tested positive for SARS-CoV-2 antigen.

The district public health officer then initiated COVID-19 public health procedures as mandated by the Thai MoPH, and the patient was transported to the district government hospital for both malaria and COVID-19 management. At the hospital, peripheral blood was drawn and tested for complete blood count (CBC), malaria blood smear, dengue NS1 (Dengue NS1 Rapid Antigen Test, Cypress Diagnostic, Belgium) and dengue antibodies (Dengue antibodies IgG and IgM, Cypress Diagnostic, Belgium), leptospirosis antibody, liver function (AST, ALT, ALP, total bilirubin, direct bilirubin, total protein, albumin, globulin), renal function (blood urea nitrogen (BUN), creatinine), and blood electrolytes (Na, K, Cl, CO_2_). Urine was also collected for urinalysis. Nasopharyngeal swab was collected to test for SARS-CoV2 by real time polymerase chain reaction (RT-PCR) at the provincial hospital in Ratchaburi, Thailand.

At admission, the patient’s vital signs were: body temperature of 37.0 °C, respiratory rate of 20 per minute (min), pulse rate of 91 per min, blood pressure of 125/114 mmHg, and oxygen saturation of 96%. He began to experience fever, cough, chills, and loss of smell (anosmia). Physical examination revealed no pharyngeal or tonsillar erythema. There was no lymphadenopathy. No hepatosplenomegaly was seen. The thoracic and neurological examinations were within normal limits. No meningeal signs were seen. His blood tests showed anemia and thrombocytopenia on complete blood counts, elevated alkaline phosphatase (ALP), and BUN with mild hyponatremia and hypochloremia. The remaining labs were within normal limits, and tests were negative for dengue NS1 Ag and IgM/IgG Ab antibodies. Full test results are shown in Table 1 and Table 2. Chest X-ray was normal (Figure S1), and a SARS-CoV-2 rapid antigen test was again positive. Moreover, *P. vivax* parasitemia (%Parasitemia = (Number of Infected Red Blood Cell/ Number of Red Blood Cell Count) × 100) was 0.35%. The patient was diagnosed with mild COVID-19 disease with *P. vivax* co-infection. Medications for the former included dextromethorphan, one tablet (tab) three times a day by mouth, loratadine 10 mg tab, once a day by mouth, and favipiravir (200 mg tab), nine tabs by mouth (PO) on the first day then four tabs for days 2–5. For malaria, chloroquine (150 mg tab), four tabs PO on day 1 and 2, then two tabs PO for day 3 was administered. In addition, radical cure primaquine (15 mg tab), one tab PO for 14 days, was prescribed with supportive care medications: paracetamol, oral rehydration solution (ORS), and intravenous fluids (5% dextrose in 0.45% normal saline solution). On day 1, within 24 h, the RT-PCR test for SARS-CoV-2 came back positive.

During the hospital stay, the patient did not require supplemental oxygen, and no other significant clinical symptoms developed. The *P. vivax* parasitemia quickly decreased to 0.05% on the second day and became negative by day 3. The patient was discharged after three days of hospitalization to a home isolation (HI) facility, to complete a 14-day quarantine and courses of primaquine and the anti-viral medication, favipiravir, for treatment of malaria and COVID-19, respectively. Vital signs at discharge were: body temperature 36.3 °C, respiratory rate of 20 per min, pulse rate of 82 per min, and blood pressure of 106/61 mmHg. The patient did not have additional SARS-CoV-2 testing after discharge, as per national guidelines at the time. 

In February 2022, the patient was found to have asymptomatic recurrent COVID-19 infection after he was tested during a close contact tracing investigation involving his family members and a neighbor. He was placed in HI for 10 days under medical guidance with telehealth visits by Suan Phueng Hospital staff. His treatment plan was identical to the first infection, including a favipiravir course with full recovery. At follow-up in late April 2022, the patient felt well and was without any signs/symptoms of long COVID-19 syndrome or recurrent *P. vivax* infection.

## 3. Discussion

This case is the first *P. vivax* and COVID-19 co-infection reported in Thailand of which we are aware and underlines the risk of malaria and COVID-19 co-infections in malaria-endemic regions, which, if not suspected or investigated, can lead to inaccurate diagnosis and treatment and/or exposure of healthcare workers. COVID-19 infection was not initially suspected due to the lack of apparent symptoms at the time of malaria diagnosis and a history of two COVID-19 vaccinations; testing was only pursued due to potential participation in a clinical study. The patient did receive chloroquine for the *P. vivax* infection, and, although there was an initial optimism for this drug (and hydroxychloroquine) to be used against COVID-19, neither has been shown to improve outcomes [7]. It could not be determined here if there was any clinical or virological effect. Since the COVID-19 diagnosis was made in late November 2021, it is likely that this represented the Delta variant, with Omicron not starting to surge in Thailand until late January–February 2022 [8]. 

Although malaria and COVID-19 both infect millions, there is limited published information to date on co-infections. A meta-analysis published in late 2021 reviewed five research articles, of which four were in Africa, and gave an overall pooled prevalence of *P. falciparum* of 5% in COVID-19 patients [9]. Hussein et al. reported a higher risk of mortality in COVID-19 patients also infected with *P. falciparum* [10]. A recent review of 600 COVID-19 patients in Uganda found 12% had *P. falciparum* co-infection [11]. Only 4% of the enrolled cohort were less than 20 years of age, but this age group had 22% of the malaria infections. History of previous malaria exposure and antibody levels to blood stage *P. falciparum* antigens both had negative associations with COVID-19 severity, even when adjusting for co-morbidities. Thailand reported only 61 *P. falciparum* cases in 2021, with 3083 cases of *P. vivax*, 72 cases of *P. knowlesi*, and 18 cases of *P. malariae* [6]; thus, understanding co-infections with these malaria species will enable comparisons to the observations from Africa.

The single clinical study in a *P. vivax* endemic region to date reported on COVID-19 infections in adult healthcare workers in India, approximately 6% of whom (27/463) had *P. vivax* malaria [12]. Interestingly, these patients had faster clearance and shorter hospital stays than the group without malaria. The authors speculated that this could be due to cross-reactive immunity, but no serology was performed as in [11]. About half the healthcare workers did take hydroxychloroquine prophylaxis for a median of 3 weeks, but the article did not comment on how often this medication was prescribed in those with malaria. 

Of the six available published case reports on *P. vivax* and COVID-19 co-infections, three are from India, one is from Indonesia, and two are from Qatar [13,14,15,16,17,18]. Five individual cases in references [13,15,16,17,18] were all male, with four being adults. Three were diagnosed with co-infection at admission, one diagnosed with *P. vivax* and turning positive for SARS-CoV-2 on day 3 of hospitalization, and one diagnosed with COVID-19 and diagnosis of *P. vivax* on day 3. While all five had fever at presentation, the constellation of accompanying acute febrile illness symptoms (headache, fatigue, myalgia, chills, etc.) was slightly different for each patient. All had thrombocytopenia with a range 30–71,000/µL. None of the patients has an oxygen saturation less than 96% on room air, none required supplemental oxygen, and all recovered from their illnesses. Four had previous histories of malaria, with three having time courses that could be consistent with relapse. In the two cases in Qatar, which does not have indigenous malaria transmission [17,18], one person had a history of malaria one year earlier when in India and one person had traveled to Pakistan three months prior, although no primary infection was recorded. A 10-year-old in India [13] had a documented episode of *P. vivax* six months prior and incomplete course of primaquine radical cure. The last case series [14] from India described three cases of *P. vivax* and COVID-19 co-infections in pregnant women, one of whom experienced fetal demise. During pregnancy, malaria is known to contribute to maternal and fetal complications [19], so it is difficult to know how much COVID-19 contributed to the outcome, but this patient was not reported to have any oxygen requirement. 

No published case reports for co-infections of COVID-19 with *P. malariae* or *P. knowlesi* were found, but three have described cases of *P. ovale*, a species also capable of relapses [20,21,22]. In all three cases, the febrile patient was first diagnosed with COVID-19, then developed *P. ovale* parasitemia 5 to 21 days later. Two cases had a history of travel from a non-endemic country (China, Turkey) to endemic countries (Uganda, Ghana), two to three weeks prior to admission. The third case of *P. ovale*, occurring in a pregnant woman in Italy, was suspected to be relapse after a trip to Burkina Faso five months earlier, but no primary infection was documented. This patient was the only one who required supplemental oxygen, but all three recovered without complications.

The burden of non-falciparum malaria, if estimated at less than 5% of all malaria cases [23], still represents roughly 10–15 million cases per year. Thus, the handful of *P. vivax* and COVID-19 co-infections reported seems to suggest it is a relatively uncommon event. In Thailand, strict public health measures and very limited international travel bans were implemented in March 2020, keeping COVID-19 cases to a minimum, with approximately only 4000 COVID-19 infections countrywide from March to November 2020 [24]. As the country began to open to travelers, larger waves then began to occur. Malaria cases in Thailand have continued to decrease during the pandemic, with 5433 cases in 2019 and 3266 cases in 2021 [6]. Thailand’s governmental strict public health policies on COVID-19, with limitations of travel and immigration across provinces and borders, as well as night-time curfews, likely had effects on mosquito exposure. In malaria-endemic areas of northeast Thailand, human mobility, as measured by radius of gyration, decreased by 90% in the first months of the pandemic [25]. Therefore, co-infections with SARS-CoV-2 may have been less prevalent in Thailand than in other countries during 2020–2021. 

There are also nascent efforts to understand if protective host factors, such as blood type or cross-reactive malarial immunity, may have reduced prevalence of COVID-19 in tropical areas [26,27]. This patient experienced two COVID-19 infections despite being vaccinated and having malaria, although both episodes were mild. Shahid et al. assessed the prior literature on the frequency of *P. vivax* and viral co-infections, reviewing 36 articles published between 1991 and 2020 [18]. Most often co-infections were with human immunodeficiency virus (HIV) or dengue; none were respiratory pathogens. There has been a case report of dengue and COVID-19 co-infection in Thailand, which occurred in March 2020 [28]. Even if malaria and viral co-infections are not common, the potential for COVID-19 infections to trigger *P. vivax* relapses needed to be evaluated closely. Relapses are known to occur after treatment for *P. falciparum* [29], but in a review by Shanks et al., *P. vivax* relapses did not appear to be more common during the 1918 influenza epidemic [30]. The case numbers published so far seem to be in line with those findings. It is possible that co-infections are underreported if patients are asymptomatic. Additionally, initially during the pandemic, COVID-19 testing may not have been widely available, or patients may have been hesitant. Guha et al. found that, when asked permission to undergo testing for SARS-CoV-2, 75% of malaria patients declined [31]. The authors also reported a decrease in malaria clinic attendance during the pandemic. As the pandemic wanes, and there is more time to synthesize and analyze the data, a more complete co-infection picture may emerge.

The proposed mechanism of reduction in morbid/mortality and cross-immunity was explained by Verner N Orish et al., with malaria-induced activation of innate immunity resulting in both trained immunity (hyperreactivity) and tolerance (hyporesponsiveness). This tolerance allows a minimal inflammatory response from innate immune cells, such as monocytes, in malaria-immune individuals, hence reducing the detrimental inflammatory effects responsible for severe malaria, which are common in individuals lacking malaria immunity. This tolerance induced by malaria may also be cross-protective, as later infections unrelated to malaria may not elicit a vigorous inflammatory response, hence preventing serious illnesses. It has been hypothesized that this tolerance may be the reason why people living in malaria-endemic regions (and possessing malaria immunity) are protected against the strong inflammatory response of COVID-19, the defining characteristic of severe SARS-CoV-2 infection [32].

Despite the overall paucity of *P. vivax* and COVID-19 co-infections reported in Thailand and Southeast Asia to date, cases could increase following the global spread of the highly transmissible Omicron variant, and, as travel and movement restrictions abate, people return to travel and forest activities. The Thai government has supported a large roll-out of affordable SARS-CoV-2 antigen test kits along with the Thailand COVID-19 national guideline training to healthcare providers. If these are consistently applied in health settings or home use, this could not only help mitigate continued community COVID-19 transmission but inform a person of the need to quarantine at home and reduce the risk of mosquito exposure and malaria infection.

## 4. Conclusions

This report of simultaneous infection with *P. vivax* and COVID-19 appears to be the first published report in Thailand and Southeast Asia. There was significant concern in 2020 for the impacts of COVID-19 on malaria, both clinically as well as from a public health perspective. The rate of malaria did increase during the pandemic in Sub-Saharan Africa, but cases remain on the decrease in Thailand. Still, as the pandemic wanes, healthcare providers in Thailand need to keep in mind a broad differential diagnosis and vigilance for both diseases, as co-infections could significantly alter the course of clinical management and prognosis.

## Figures and Tables

**Table 1:** Complete blood count and additional laboratory results at admission.

Laboratory Test	Result	Normal Range
Hemoglobin (Hb)	11.5	13.8–17.2 g/dL
Hematocrit (Hct)	34	40.7–50.3%
Red blood cell count	4.27	3.50–5.50 × 10^6^ cell/uL
White blood cell count	4190	4000–10,000 cell/cu.m
Platelet count	92,000	140,000–400,000 cell/cu.m
Mean corpuscular volume (MCV)	80.4	80–100 fL
Mean corpuscular hemoglobin (MCH)	26.9	27–31 pg
Mean corpuscular hemoglobin concentration (MCHC)	33.5	31–37 g/dL
Neutrophil	59	50–70%
Lymphocyte	21	20–40%
Monocyte	16	3–8%
Eosinophil	2	0–5%
Basophil	0	0–2%
Atypical lymphocyte	2	0
Red blood cell morphology	Normal	Normal
Malaria (Thin–Thick film)	Positive	
Malaria type	*Plasmodium vivax*	
Parasitemia	0.35%	
SARS-CoV2 Real time RT-PCR	Detected	
Nasopharyngeal swab	N gene Ct = 27.01	
	ORF1 ab gene Ct = 25.94	
Dengue NS1	Negative	
Dengue Ab,IgM	Negative	
Dengue Ab,IgG	Negative	
Leptospira Antibody	IgM weakly positive	

**Table 2:** Blood chemistry results at admission.

Laboratory Test	Result	Normal Range
Blood urea nitrogen (BUN)	16	7–25 mg/dL
Creatinine	0.68	0.70–1.17 mg/dL
e-GFR	132.73	97.00–137.00 mL/mon/L
Sodium (Na)	133	135–145 mmol/L
Potassium (K)	3.6	3.5–5.1 mmol/L
Chloride (Cl)	93	99–111 mmol/L
Carbon dioxide (CO_2_)	24	22–33 mmol/L
Total Protein	6.8	6.0–8.3 g/dL
Albumin (Blood)	3.5	3.2–5.2 g/dL
Globulin	3.3	2.0–3.0 g/dL
Total Bilirubin	1.6	0–2 mg/dL
Direct Bilirubin	0.6	0–0.2 mg/dL
Aspartate transaminase (AST)	22	0–35 U/L
Alanine transaminase (ALT)	42	0–45 U/L
Alkaline phosphatase (ALP)	133	53–128 U/L

## Data Availability

The data presented in this study are available on request from the corresponding author.

## Supplementary Materials

The following supporting information can be downloaded at https://www.mdpi.com/article/10.3390/tropicalmed7080145/s1. Figure S1: Patient’s Chest X-ray on the date of admission.
