# Supplementary figures and images for: Co-Infection with Plasmodium vivax and COVID-19 in Thailand
Source: Trop Med Infect Dis. 2022 Jul 22;7(8):145. doi: 10.3390/tropicalmed7080145 (PMC9332623; doi:10.3390/tropicalmed7080145)

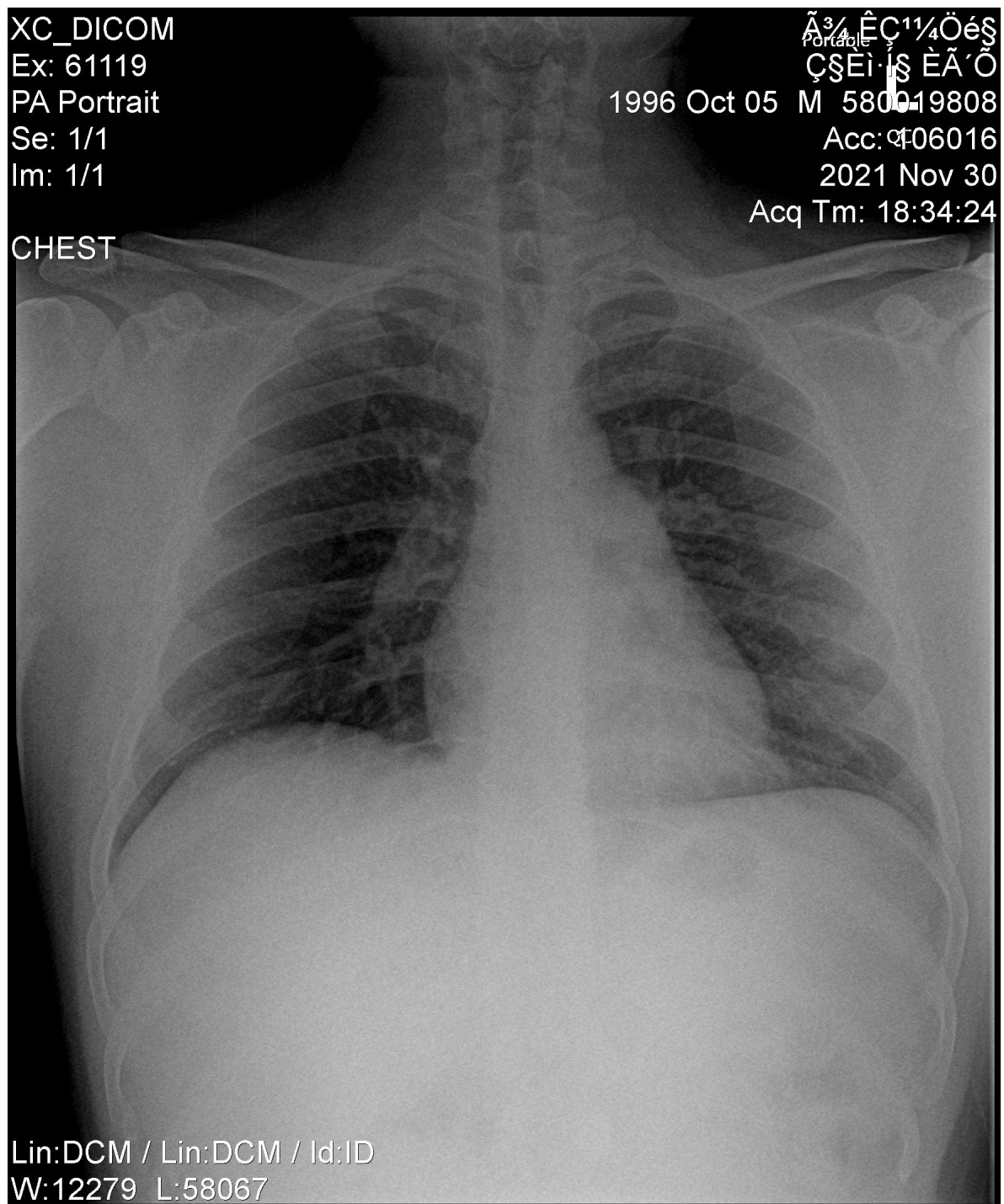

**Figure S1.** Patient's Chest X-ray on the date of admission.

Supplement: Supplementary file 1 [file tropicalmed-07-00145-s001.zip › tropicalmed-1805739-SI.pdf]
